# Quantitative CT imaging and advanced visualization methods: potential application in novel coronavirus disease 2019 (COVID-19) pneumonia

**DOI:** 10.1259/bjro.20200043

**Published:** 2021-01-25

**Authors:** Prashant Nagpal, Junfeng Guo, Kyung Min Shin, Jae-Kwang Lim, Ki Beom Kim, Alejandro P Comellas, David W Kaczka, Samuel Peterson, Chang Hyun Lee, Eric A Hoffman

**Affiliations:** 1 Department of Radiology, University of Iowa, Carver College of Medicine, Iowa City, IA, USA; ^2^ Roy J. Carver Department of Biomedical Engineering, University of Iowa, College of Engineering, Iowa City, IA, USA; ^3^ Department of Radiology, School of Medicine, Kyungpook National University, Daegu, South Korea; ^4^ Department of Radiology, Daegu Fatima Hospital, Daegu, South Korea; ^5^ Department of Internal Medicine, University of Iowa, Carver College of Medicine, Iowa City, IA, USA; ^6^ Department of Anesthesia, University of Iowa Carver College of Medicine, Iowa City, IA, USA; ^7^ VIDA Diagnostics, Coralville, IA, USA; ^8^ Department of Radiology, Seoul National University College of Medicine, Seoul National University Hospital, Seoul, South Korea

## Abstract

Increasingly, quantitative lung computed tomography (qCT)-derived metrics are providing novel insights into chronic inflammatory lung diseases, including chronic obstructive pulmonary disease, asthma, interstitial lung disease, and more. Metrics related to parenchymal, airway, and vascular anatomy together with various measures associated with lung function including regional parenchymal mechanics, air trapping associated with functional small airways disease, and dual-energy derived measures of perfused blood volume are offering the ability to characterize disease phenotypes associated with the chronic inflammatory pulmonary diseases. With the emergence of COVID-19, together with its widely varying degrees of severity, its rapid progression in some cases, and the potential for lengthy post-COVID-19 morbidity, there is a new role in applying well-established qCT-based metrics. Based on the utility of qCT tools in other lung diseases, previously validated supervised classical machine learning methods, and emerging unsupervised machine learning and deep-learning approaches, we are now able to provide desperately needed insight into the acute and the chronic phases of this inflammatory lung disease. The potential areas in which qCT imaging can be beneficial include improved accuracy of diagnosis, identification of clinically distinct phenotypes, improvement of disease prognosis, stratification of care, and early objective evaluation of intervention response. There is also a potential role for qCT in evaluating an increasing population of post-COVID-19 lung parenchymal changes such as fibrosis. In this work, we discuss the basis of various lung qCT methods, using case-examples to highlight their potential application as a tool for the exploration and characterization of COVID-19, and offer scanning protocols to serve as templates for imaging the lung such that these established qCT analyses have the best chance at yielding the much needed new insights.

## Introduction

Coronavirus disease 2019^1^ has taken the world by storm. The disease is caused by a novel strain of coronavirus, Severe Acute Respiratory Syndrome Coronavirus 2 (SARS-CoV-2). The virus primarily affects the respiratory system and manifests as pneumonia, which frequently can be seen on imaging, especially CT.^2,3^ The disease started from mainland China in 2019, and by the end of January 2020 was declared as a global health emergency by the World Health Organization.^4,5^ The disease has highlighted the lack of preparedness of health care to deal with a new and emerging pandemic.^6,7^ The therapeutic options, long-term effects, and methods to follow up the disease are still being studied. Given the public health concerns with the disease, any step that can help understand disease pathogenesis, improve the diagnosis, prognostication, or management of the disease will be critical. Currently, there are significant challenges and questions pertaining to the diagnosis and management of the disease. Some of the challenges include the choice of test for screening and diagnosis of the disease (especially when the reverse transcriptase-polymerase chain reaction (RT-PCR) tests have not been readily available or were unreliable), assessment of disease severity, triage of patients for a hospital *vs* home care, appropriate prognostication of individual patients, and assessing early response to therapy.^8^ A recent statement from the Fleischner Society^9^ has highlighted that key roles for imaging are to identify patients at risk of disease progression and to help triage moderate-to-severe cases for hospital *vs* home care. With PCR availability and accuracy improving, at this stage of the pandemic (as of October 2020), tools are needed that allow better characterization of the lung disease phenotypes leading to an enhanced understanding of the lung pathology and variable patient prognosis. Hence, in this practice and policy paper, we outline a set of well-utilized set of quantitative lung CT (qCT) metrics that can be applied to COVID-19 if basic principles of qCT scanning are followed.

We would add that, as we seek to identify interventions, there is a need for tools to detect successes early in the course of the disease rather than waiting to treat late. In addition, early studies have demonstrated that imaging will likely play an important, retrospective role in understanding the unique phenotypes of this disease^10^ as well as understanding the residual pathologies upon recovery.^11^

Understanding of the role of CT imaging in the disease is evolving. Initial reports suggested that chest CT may be more sensitive than the current tests of choice, such as RT-PCR.^12^ These reports suggested that CT should be used for screening in symptomatic or suspected patients, but such an observation may be related to a lack of technical expertise with RT-PCR. Additionally, CT imaging can be very non-specific with regard to COVID-19, with features overlapping with other pathologies, such as influenza, bacterial pneumonia, or inflammatory drug reactions. Factors currently inhibiting the use of CT are concerns, which include poor imaging room ventilation, relative lack of personal protective equipment, difficult logistics involved in patient transportation to the CT facility, and the need for lengthy room disinfection after testing. Hence, major radiology organizations worldwide (American College of Radiology [ACR], Society of Thoracic Radiology, and American Society of Emergency Radiology [STR and ASER], British Society of Thoracic Imaging [BSTI], The Royal Australian and New Zealand College of Radiologists [RANZCR]) have suggested that CT should not be used for screening or diagnosis if reliable RT-PCR testing is available. The role of CT should be reserved for hospitalized, symptomatic patients with specific clinical indications for CT, and a portable chest radiograph should be preferred in the suspected patients.^13–16^ However, the clinical manifestations frequently overlap with other diseases for which CT imaging is usually performed (*i.e.* chest pain, shortness of breath, fever in immunocompromised patients, rule-out pulmonary embolism etc.), and both chest CT and radiographic imaging are being performed among COVID-19 suspected or confirmed cases. As putative interventions emerge, qCT can provide a better understanding of disease pathogenesis and an early objective assessment of outcomes, allowing for continuance of the intervention or early termination.

The methodology of qCT allows for automated, reproducible, and quantifiable metrics that can measure normal and diseased lungs on CT images. Quantitative lung CT metrics have been primarily studied and validated for chronic lung diseases and, compared to a conventional review of images or pulmonary functional tests (PFTs), have been shown to provide a better characterization of distribution and percent of the lung affected by the disease^17^; disease prognostication^18^; prediction of worse clinical outcomes and mortality^18^ ;as well as objective monitoring of early response to therapy.^19,20^ Quantitative lung CT applications in inflammatory lung disease have included, for instance, chronic obstructive pulmonary disease (COPD),^21–25^ interstitial lung disease (ILD),^26–28^ or lymphangioleiomyomatosis (LAM).^29,30^ However, qCT metrics have not been as well studied in acute lung diseases. Pulmonary applications of qCT in association with COVID-19 are a novel application, allowing the following of rapidly changing pulmonary disease processes. Quantitative features that can be applied to lung CT images are based on lung density, texture, and airway or vascular mapping.^28,31,32^ These advanced lung imaging tools have been developed using a consistent imaging protocol that has been validated and applied in various multicenter studies.^33^ The use of these validated advanced lung imaging tools can help better quantify the disease burden and may help triage the patients. Additionally, as COVID-19 patients with comorbidities or baseline lung disease^34^ have a poor prognosis and are at higher odds for severe disease, qCT can help by objectifying the characterization of the underlying disease phenotypes associated with, *e.g.* COPD, asthma, ILD and most certainly have a new role to play in the acute and potentially more chronic state of the lung associated with COVID-19. These tools can help with better patient prognostication and aggressive management, when needed. In this practice and policy document, we highlight the applications of advanced lung imaging tools as applied to COVID-19 disease and their potential role. With the desire for objective quantification comes the need for standardizing imaging protocols to ensure accuracy of the resulting metrics and to allow for harmonization of image findings across centers,^33^ which in turn will allow for the application of emerging technologies such as deep learning.^35–38^

### Current status of artificial intelligence (AI) and machine learning-based tools

As with the understanding of the disease itself, the role of AI (*e.g.* classical ML or deep learning methodologies) for COVID-19 pneumonia is evolving by utilizing the quantitative information from CT images and submitting these to various computer-based processes. Radiomics is a methodology that extracts a large number of features from imaging data that would not otherwise be apparent to visual inspection. Radiomics uses a data-characterization algorithm to bin the features into clusters that are most relevant for a specific clinical context. Such processes yield new insights into distinct disease phenotypes, which in turn can provide for an understanding of underlying pathology. A recent position statement by RANZCR briefly addresses this topic and highlights that AI research and technological imaging advances for their added role in the clinical care of patients should be supported.^15^ A few critical but very important points highlighted in the position statement are that quantitative image analysis tools should add value to visual image inspection by providing a better understanding of the disease rather than simply targeting a reduction in workload or turnaround times as those are currently not the limiting factors. Another vital step towards the usefulness of quantitative image analysis tools is generalizability to different population groups and integration into the existing clinical practices.

In the current stage of the pandemic (as of October 2020), there is increasing utilization of CT imaging in patients with COVID-19 pneumonia. Based on the experience with the use of quantitative analysis tools for multiple other lung pathologies, there is a potential for valuable applications in COVID-19 disease. The initial reports on applications of AI-based tools for COVID-19 disease are promising.^5,39–42^ Some of the deep learning-based methods are focused on improving the sensitivity or diagnostic accuracy for COVID-19, which are new and have scant (if any) validation data for their generalized or wide-spread applicability.^5,39,43^ In this work, we will focus on established qCT methodologies that have been validated and utilized in various multicenter studies in collaboration with our research laboratory over last two decades.^31,32,44–51^ These methodologies have been combined with various statistical and other machine-learning analyses, imparting added insights into lung diseases. If appropriately used, these methods can provide direct insights into the disease severity, distribution, progression, and regression as well as identify underlying variations (sub-phenotypes such as parenchyma *vs* vasculature) in pathology, which may not be otherwise apparent. The development and testing of the generalizability of these tools require the use of standardized imaging protocols. By organizing such commonality of imaging practices, we have an opportunity to utilize this crisis to bring radiology fully into the emerging era of computer-assisted objective, quantitative image analysis.^52^

### Quantitative lung CT imaging tools

#### Density and histogram-based disease quantification

The attenuation values of voxels on the lung CT vary between −1000 Hounsfield units (HUs) (air), 0 HU (water), and +1800 HU (cortical bone) and is related to the tissue characteristics. Various lung diseases can be objectively measured using quantification of voxel attenuation. The image feature to be quantified is based on the type of abnormality on the CT image (low attenuation area [LAA] *vs* high attenuation area [HAA]). Because the lung is composed essentially of air and tissue with just two HU values, the HU value of a voxel allows one to determine the percent air and percent “tissue” (blood, tissue, and extracellular fluid).^53,54^ As regional inflammation progresses, voxel “tissue” volume increases. Thus, short-term (weeks or months) longitudinal changes in tissue volume can serve as an index of progression or regression of inflammation, since it would not be expected that actual parenchymal volume would change acutely. Emphysema quantification is performed by measuring the LAAs. Initially, for emphysema quantification, a cut-off HU unit value of −910 HU was suggested (areas having an attenuation of −910 HU or less characterized as emphysema).^55^ It has now been demonstrated that the cut-off value of −950 correlates better with the pathological finding of emphysema,^56,57^ while −910 HU^58^ captured emphysema at earlier stages of the pathology. Other studies suggest that a threshold of −960 or −970 HU can be used^59,60^; the differentiation between these thresholds is mostly immaterial.^61^ One needs to remain consistent, and −950 HU for advanced disease and −910 HU for early disease processes remain widely accepted.^62,63^ CT has been used to identify the COPD phenotypes which were not identifiable by the diagnostic standard PFT. COPD phenotypes identifiable by CT are also demonstrated to have similar clinical symptoms and prognosis,^63,64^ and the presence of emphysema-like voxels in subjects with normal PFTs has been found to be a predictor of future PFT changes. When quantified, the degree of emphysema is associated with poor exercise capacity,^63^ have poor overall health-status,^65^ and higher mortality.^66^ Identification of COPD phenotypes have also allowed management of individuals based on patterns of disease that are only identifiable by CT.^67^ While attenuation values of less than −950 HU helps to quantify emphysema, quantification of the lung density voxel histogram has been demonstrated to characterize fibrotic lung processes^68^ and areas with attenuation between −250 and −600 (HAAs) has been used to identify fibrotic-like lung.^69^ Because normal lung has some voxels below −950 HU and voxels between −250 and −600 HU, large population-based studies have been used to establish normative equations taking into account sex, age, race, scanner make and model etc. to provide a threshold above which one can consider an individual as having the presence of emphysema or fibrosis.^21,70^ This has been extensively studied in the context of interstitial lung diseases, where the HAA corresponds to the areas of inflammation and fibrotic lung processes.^71–73^ These HAA are also shown to correlate with worse patient health status and poor outcomes.^71,72,74,75^ In the MESA Lung study,^73^ subjects with HAA areas on CT were found to have higher serum inflammatory markers, poor respiratory function, and increased overall mortality. However, due to physiological changes in the lungs with inspiration and expiration, adherence to a standard imaging protocol is imperative to allow disease quantification and prevent errors due to variability in interpretation by the radiologists. Studies have shown that submaximal inspiration leads to an underestimation of emphysema^76^ and overestimation of HAA.

**Figure 1.**
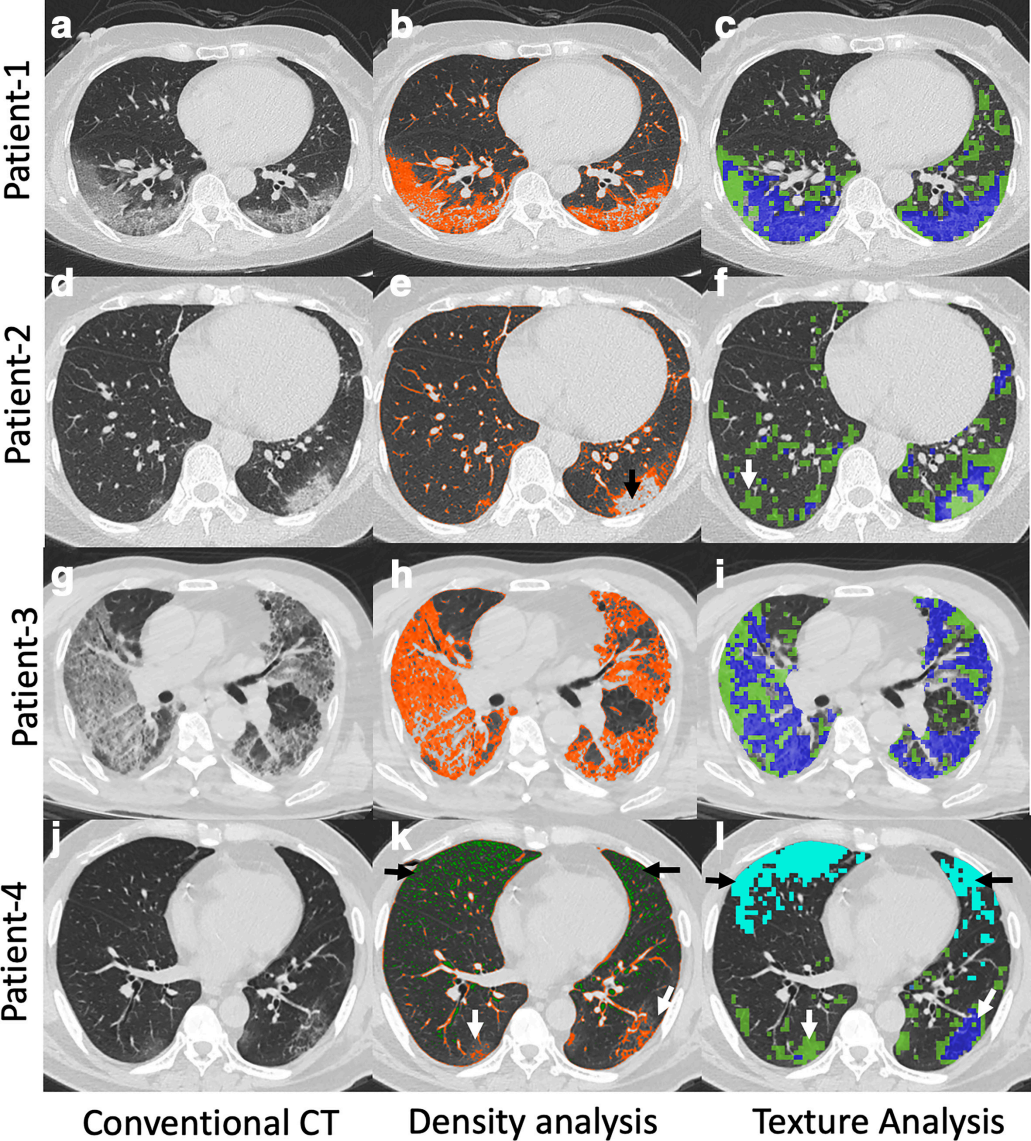
Conventional CT (first row), lung density (second row), and 3D AMFM Texture (third row) quantification in four patients with COVID-19 pneumonia. Patient 1: 67-year-old female with conventional CT (a) demonstrating posterior bilateral lower lobe mixed GGO and interstitial thickening. Density analysis (b) demonstrating HAAs (orange color) which was quantified as 14%. 3D AMFM Texture analysis (c) demonstrating abnormal lung (green color = GGO; blue color = GGO with reticular thickening) which was quantified as 27% (GGO,16%; GGO with reticular thickening, 11%). Patient 2: 46-year-female with conventional CT (d) demonstrating left more than right high-attenuation posterior GGO. Density analysis (e) underestimated the disease (quantified as 8%) because of relatively high attenuation in the central portion of the abnormality (black arrow). However, 3D AMFM Texture analysis (f) quantified the entire extent of disease, characterized it as predominantly GGO (green, 15%) with intermixed areas of interstitial thickening (blue, 3%), and also highlighted certain areas in the right lung (white arrow) that did not have identifiable disease on conventional CT. Patient 3: Adult male patient with COVID-19 pneumonia, conventional CT (g) demonstrating extensive bilateral disease with intermixed areas of GGO and interstitial thickening. Density analysis (h) showing HAA (orange color) quantified as 46% and 3D AMFM Texture analysis (i) quantifying the amount of GGO (green color, 31%) and mixed GGO and interstitial thickening (Blue, 35%). Patient 4: Conventional CT (j) demonstrating bilateral lower lobe GGO intermixed with areas of interstitial thickening with disease more in left. No abnormality is appreciated in the anterior lung on conventional CT. Density analysis (k) showing HAA (orange, white arrows) quantified as 6% with low attenuation areas in the anterior lung (green color in K, black arrows, 5%). 3D AMFM Texture analysis (l) also demonstrated abnormality corresponding to a region of hyperlucency (light blue, black arrows, 10%) in the anterior lung with better quantification of disease (GGO 8%, GGO with reticular thickening 2%) in the posterior lungs as compared to density analysis (white arrows in L). hyperlucent areas, which visually do not appear to be emphysematous, (detected as a texture abnormality) are possibly due to microemboli-induced reduction in regional blood volume. Despite relatively less lung involvement, this patient had a protracted ICU course and died on Day 25. AMFM, adaptive multiplefeature method; GGO, ground glassopacity; HAA, high attenuation area.

In patients with COVID-19 pneumonia, bilateral patchy peripheral ground glass opacity (GGO) and consolidation are typical findings.^77^ These areas are represented by HAAs,^78^ which have been validated for quantification and utility in various studies on lung diseases, especially interstitial pulmonary fibrosis. HAA can be quantified using a density- or histogram-based technique with a threshold of −250 to −600 HU (Figure 1, middle column). As in other lung diseases, disease quantification may allow objective determination of disease severity, better patient prognostication, and can also help with patient management. This tool can be used for objective quantification of the disease and can also help in finding cut-offs for disease severity on imaging that can complement clinical assessment to triage patients for care strategies during the current pandemic and in the future. Some early studies using density-based disease quantification have shown promise. In a single-center study of 262 patients with COVID-19 infection,^79^ the quantification of the compromised lung (defined as lung with HU between −500 and +100 HU) showed a significant correlation with the need for oxygenation support, intubation, and in-hospital death. In this study, it was also demonstrated that the %compromised lung between 6 and 23% was associated with oxygenation therapy, >23% was associated with the need for intubation, and the quantification of disease lung had a negative correlation with PaO2/FiO2 ratio on arterial blood gas (ABG) analysis. In another 79 patient study,^80^ quantification of lung inflammation on CT (cut-off value >14.2%) correlated with decreased PaO2/FiO2 ratio based on ABG. In another study with 236 patients with COVID-19 infection,^81^ automated density-based quantification of the well-aerated lung (WAL, defined as lung with HU between −950 and −700 HU) was performed. A higher percentage of WAL correlated with better clinical outcomes, and lower WAL correlated with significantly higher odds of ICU admission and death. When quantification of WAL from CT was added to the clinical prognostication markers, the diagnostic performance of the models significantly improved. These early feasibility studies highlight the potential of CT-based disease quantification for triage and prognostication of patients with COVID-19 pneumonia.

### Texture analysis

Computational methods for texture have been developed, and their utility has been analyzed in multiple diseases, including COPD, ILD, and lung cancer. CT-based texture assessment is automated and can help differentiate between normal and abnormal lung quantitatively. Furthermore, it can characterize and quantify the abnormality into various disease or imaging patterns. Texture-based methods make use of dozens of mathematical formulations of grayscale patterns within the CT image, and can be used in combination with various forms of supervised or unsupervised machine learning to reduce a regional multidimensional set of textures down to several textures serving to define a parenchymal classification. Some of the expected patterns which AI-based texture software can be trained to classify include: normal aerated lung, normal bronchovascular bundles, GGO, mixed ground-glass and reticular disease, consolidation, and honeycombing. Quantitative lung CT-based texture analysis can be obtained on a two-dimensional (2D) or a three-dimensional (3D, isotropic voxels) data set. An earlier 2D texture-based classification method applied to the lung was the adaptive multiple feature method (AMFM), which utilized observer assigned classifications to 2D regions of interest combined with a Bayesian classifier to convert combinations of texture types into a parenchymal descriptor.^17,82,83^ This form of development of automated learning methodology is known as ‘supervised’ ML. Such a development relies on a standardized training set and consistent labeling of the normal and diseased lung. The 2D texture analysis was later extended to a more sensitive and specific 3D AMFM method.^84,85^ CT texture-based analysis has been shown to identify and differentiate, *e.g.* normal lung, respiratory bronchiolitis, emphysema, and honeycombing. There is increasing evidence in support of CT texture-based methods for investigating clinical questions. Among studies of smoking subjects, 3D AMFM texture-based classification has, as an example, been shown to differentiate a non-smoker and a smoker lung from a pool of subjects with normal PFT.^85^ For COPD evaluation, various multicenter research studies have utilized texture methodologies to objectively characterize parenchymal pathologies and correlate them with outcomes.^74,86–89^ For interstitial lung diseases, texture-based quantification of imaging abnormalities has been shown to be useful to predict disease progression among patients with idiopathic pulmonary fibrosis.^18^

**Figure 2.**
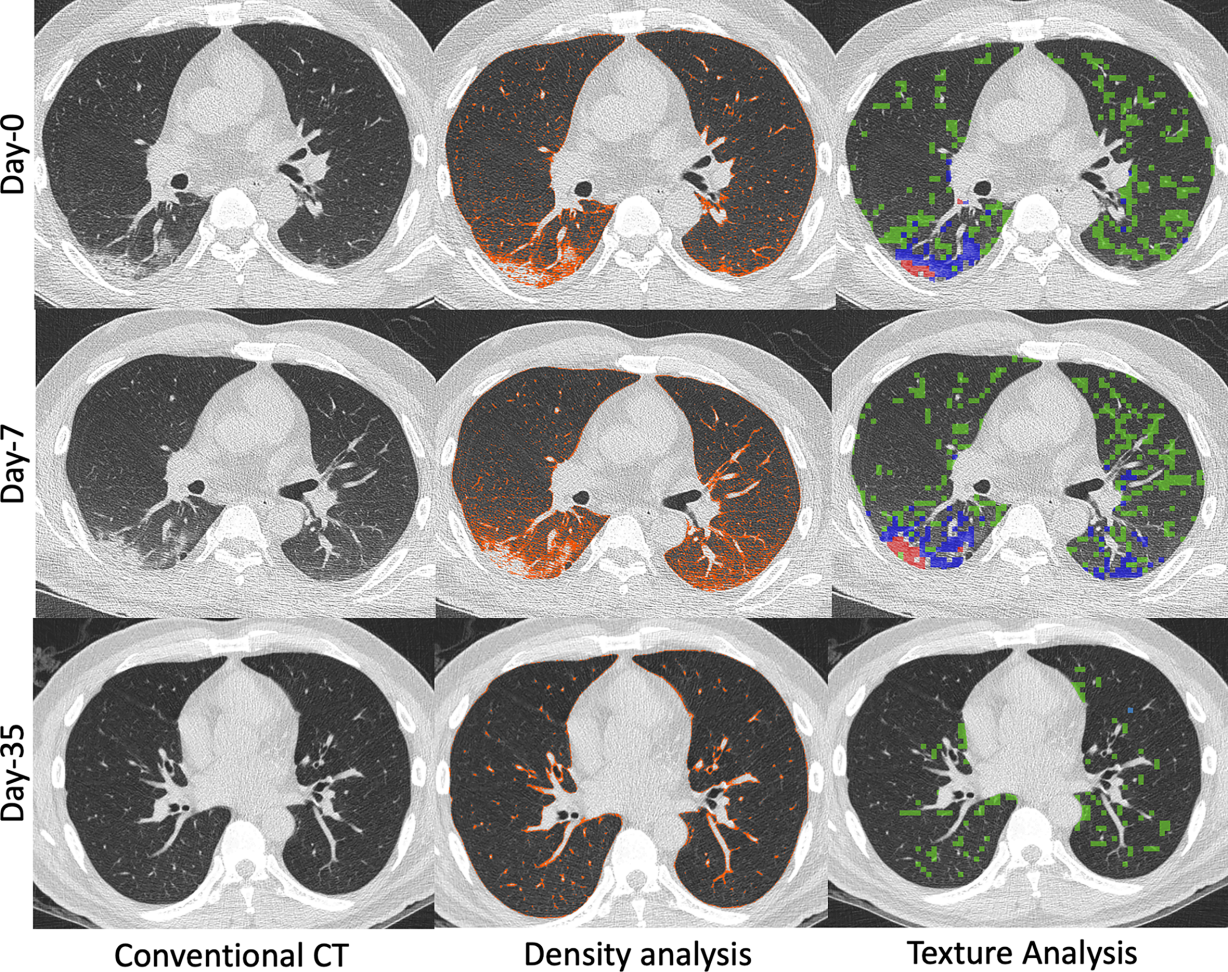
55-year-old male with COVID-19 pneumonia that received imaging at presentation (Day 0), Day 7 of hospitalization, and Day 35 of hospitalization. Images generated at initial presentation (Top row, Day 0) along with computed density and texture analysis demonstrating predominantly posterior bilateral lower lobe disease (HAAs quantified as 13% on density analysis and abnormal lung quantified as 24% on texture analysis). The texture analysis characterized the disease as GGO (green, 21%), mixed GGO and interstitial (blue, 3.9%), and consolidation (red, 0.1%). Follow-up CT (Middle row, Day 7) obtained due to worsening shortness of breath demonstrated minimal disease progression on conventional CT images and density analysis and texture analysis provided objective assessments of disease worsening (HAA on density analysis quantified as 18% and abnormal lung quantified as 29% on texture analysis). Progression of imaging disease type was also quantified (GGO, green: 22.5%; mixed GGO and interstitial, blue: 6.3%; and consolidation, red: 0.2%). Another follow-up CT (Bottom row, Day 35) obtained after clinical improvement and prior to discharge from the hospital demonstrated complete resolution of disease on conventional images. The density and texture analysis identified some residual abnormalities (HAA 5% on density analysis and abnormal lung 6% on texture analysis). GGO, ground glassopacity; HAA, high attenuation area

In patients with COVID-19 pneumonia, texture analysis can differentiate normal from abnormal lung (providing a score representing the percentage of the normal lung) by characterizing abnormal lung as various imaging patterns such as GGO, mixed GGO/interstitial, and consolidation (Figure 1, right column). Of interest, a portion of this patient’s ventral lung was labeled by the AMFM as having “emphysema-like” features. Visually, the lung did not appear emphysematous, but the density measurements fell below −950 HU. It is possible that such regions (also observed in other COVID patients) may have a reduced regional blood pool related to emerging reports of microemboli.^90–92^ Such examples suggest that CT texture analysis in COVID-19 patients may help quantify the imaging patterns and potentially explain low arterial oxygenation when visual assessments of disease extent fail. These observations may help identify distinct disease phenotypes (*i.e.* lung parenchymal disease *vs* vascular disease). As depicted in Figure 2, both texture analysis and density-based methods provide for an objective, quantitative assessment of progression or regression of disease. As can be seen in both Figure 1 and Figure 2, the density-based method misses the inclusion of dense consolidation with attenuations greater than −250 HU. However, if the threshold range is expanded to include the consolidation, normal bronchovascular bundles are included with the regions of pathology, leading to overestimation of the size of diseased regions. This is avoided by the use of the texture-based approach. As demonstrated in Figure 2, texture can be used to quantify changes in parenchymal involvement over time, offering a sensitive quantitative assessment of disease progression, regression, and, thus, response to therapy even when such changes are not apparent on subjective visual review of images. In this particular example, in addition to quantitation of texture types, the change in the non-air volume of the lung serves as a measure of changing inflammatory burden.

### Summary or topographic multiplanar reformatted views

**Figure 3.**
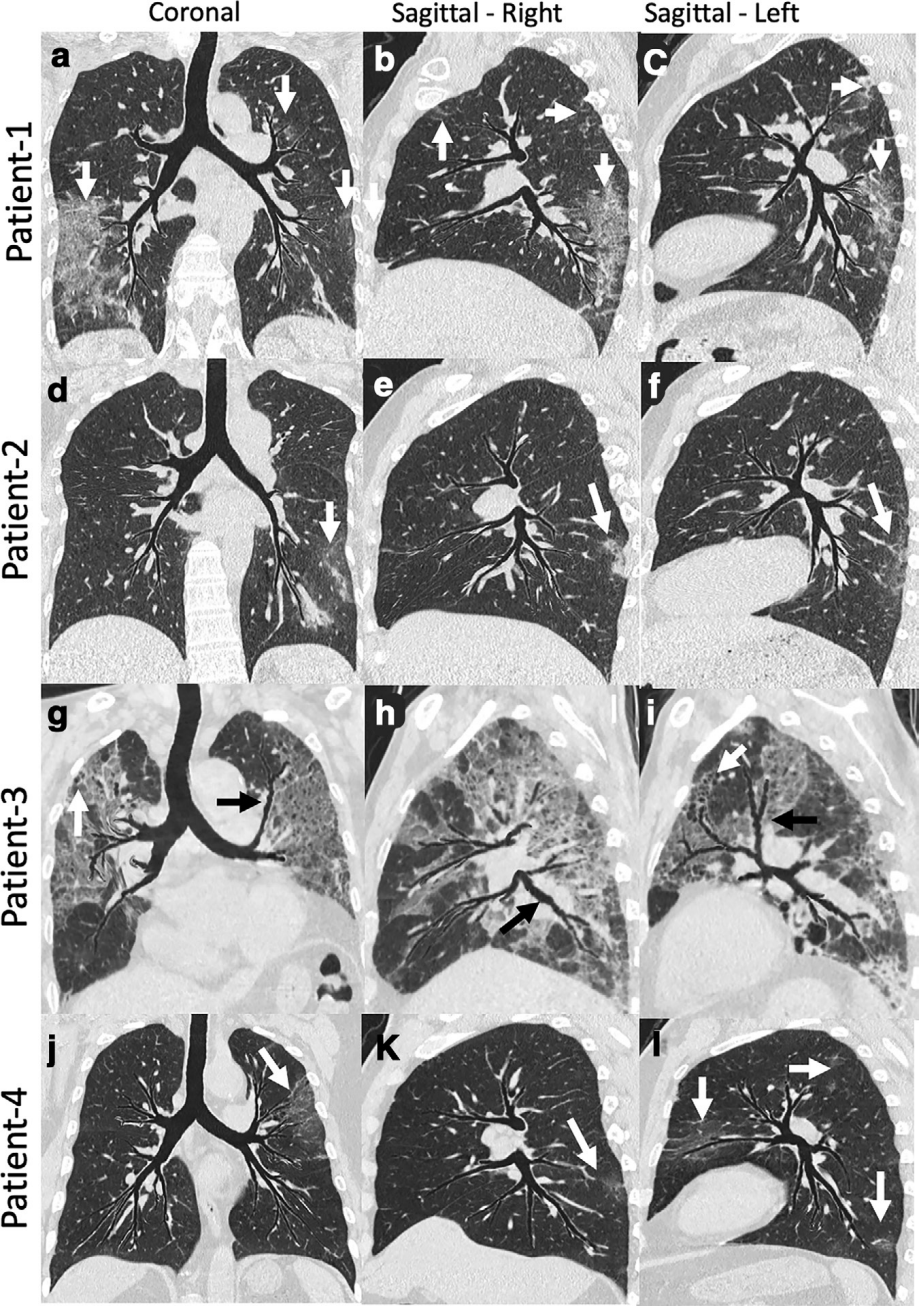
tMPR view images of four patients with COVID-19 pneumonia. The tMPR view warps the airway tree such that most major subsegmental airway segments reside within a single flattened projection, and associated parenchyma and vasculature are similarly warped. Patient 1: Coronal (a) and Sagittal (**b, c**) images showing multifocal, predominantly peripheral bilateral ground glass opacification (white arrows). These images provide comprehensive visualization of airway and overall extent of disease. Patient 2: Coronal (d) and sagittal (**e, f**) images showing bilateral peripheral mixed ground glass opacity and reticular thickening (white arrows). The coronal image demonstrates left-sided disease, but the right sagittal image demonstrates posterior lower lobe disease as well. Patient 3: Coronal (g) and sagittal (**h, i**) images demonstrating extensive bilateral upper and lower lobe disease. The patient had a protracted course and the imaging was obtained later in the time course. Images show bilateral central bronchiectasis (black arrows) and distal bronchiolectasis (white arrow). Patient 4: Coronal (j) and sagittal (k, l) images demonstrate multifocal (white arrows) left-side-predominant disease. Review of these three images provide a good appreciation of localization and the relationship with the airway paths. tMPR, topographic multiplanar reformatted.

**Figure 4.**
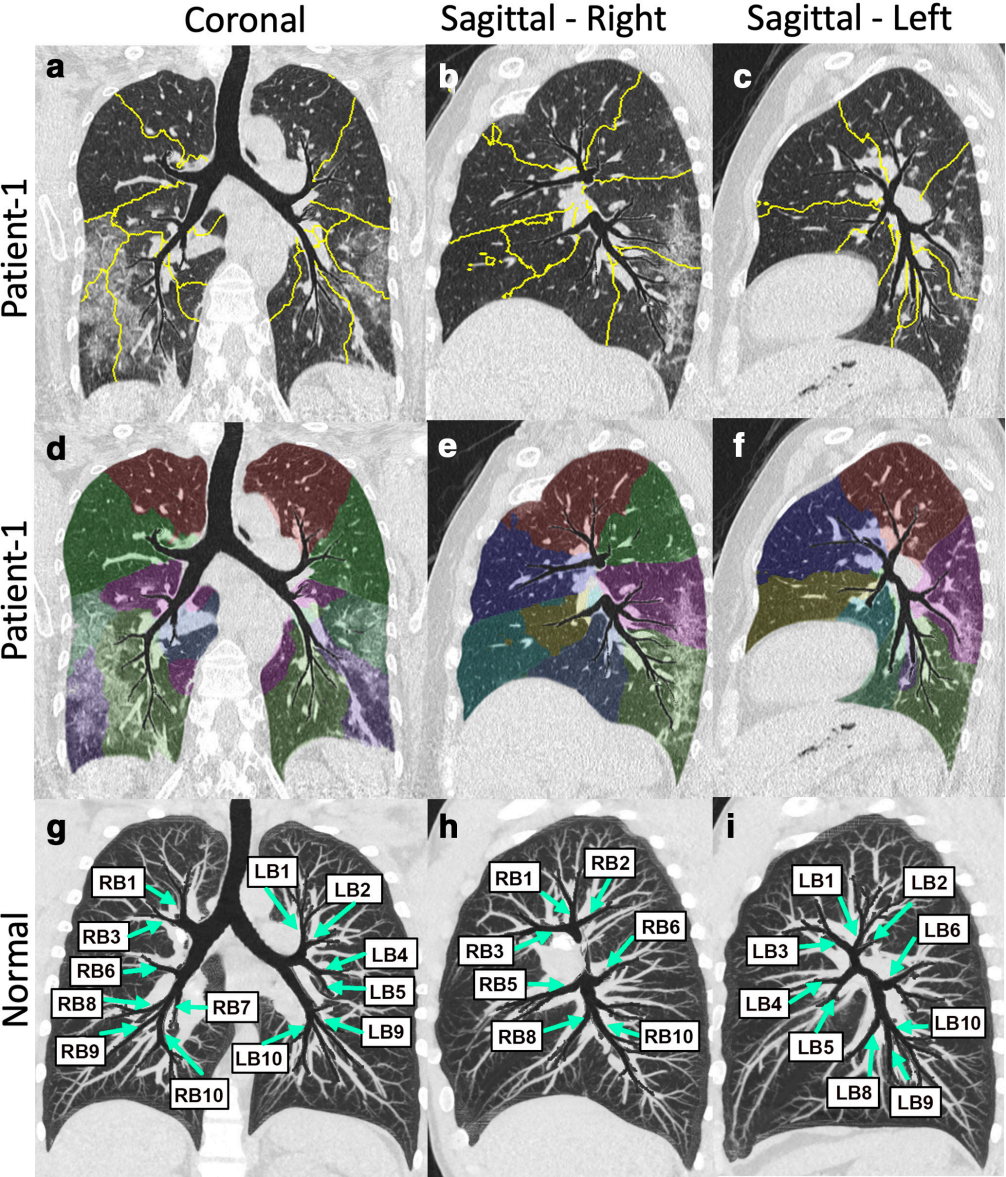
AI-based delineation of sublobar segments based on regions of influence of the airway branches. Shown here are the tMPR views of Patient 1 shown in Figures 1 and 3. Top row coronal (a) and sagittal (**c, **d) images showing outlines of the sublobar segments within the tMPR views. Middle row again shows the coronal (d) and sagittal (**e, **f) oriented tMPR views highlighting the sublobar segments via use of transparent color coding. Bottom row shows tMPR view of a normal subject. The views are thickened by use of a maximum intensity projection with regions of the airways protected from overlapping structures. This serves to put the airways and vessel branching in context of each other. By labeling the airway segments, we emphasize that the full segmental tree has been included within a single, flattened view. AI, artificial intelligence;tMPR, topographic multiplanar reformatted.

While the review of conventional CT images (axial, sagittal, and coronal) is paramount for the diagnosis of lung injury, certain advanced image visualization techniques have the potential to help with the interpretation and improve the turn-around-time in challenging times like this. Topographic multiplanar reformatted (tMPR)^93^ views, (VIDA Diagnostics, Coralville, Iowa) provide a comprehensive view of the airway tree and associated parenchyma in a single plane. These are developed using a tMPR technique that warps and flattens non-overlapping airways and their local environment. The warped parenchymal display can be further thickened into a maximum intensity projection (MIP).^94^ The tMPR views are different from conventional MPR views (commonly generated at imaging workstations) as the tMPR view flattens the airway tree along with associated parenchyma, highlighting the association between affected parenchyma and the feeding bronchial path. This is not achieved with conventional MPR techniques. Unlike the MPR, the tMPR view requires a series of automated but complex processing steps, including airway segmentation and branching structure extraction. By capturing mid-coronal as well as left and right sagittal views this way, the tMPR views allow the review of typical bronchocenteric imaging patterns,^77^ as well as the location of disease in one coronal and two sagittal snapshots (Figure 3). Additionally, this technique can be applied to the quantification of the disease presence within the individual sub lobar segments of the lungs (Figure 4), enabling objective metrics related to functional compromise and the means for follow-up, with important implications for subphenotyping and for the assessment of disease interventions. The technique thus has the potential to assess the correspondence of parenchymal *vs* airway pathology. Such displays, as has been seen with simple MIP or other sliding slab displays,^95^ may also improve the interpretation efficiency, and can be useful for rapid evaluation of changes over time using serial scans for disease comparison. Unlike other thick or sliding slab views, the tMPR view lays out a significant portion of the airway tree and its associated parenchyma in a single flattened depiction. In Figure 4, note that all of the major segmental airways are present within a single coronal view. Similarly, the right or left sagittal oriented views demonstrate the full set of right or left segmental airways.

### Inspiratory and expiratory imaging for assessment of functional small airways disease (fSAD) representing regional air trapping

Due to the difficulties of breath-holding at low lung volumes, expiratory imaging may not be possible in acutely ill patients. However, it offers utility in the post-COVID-19 assessment of residual lung damage. Parametric response mapping (PRM)^96^ and disease probability map (DPM)^97^ utilize image registration to map inspiratory and expiratory images together. In emphysema, the PRM has been useful to identify the regions of air trapping on full expiration based on density thresholds which, may not be identified from an image obtained at full inspiration. The DPM also uses image matching between full inspiration and full expiration to identify hyperinflated regions and regions of fSAD. However, the warping function must be used to characterize regional volume changes together with the density threshold to identify regions of fSAD. In acute respiratory distress syndrome (ARDS) and other forms of acute lung injury requiring invasive mechanical ventilation, the PRM has been shown to help identify regions at increased risk for atelectrauma and ventilator-induced lung injury, based on voxels transitioning between high and low density during a breath.^98^ Because of the highly contagious nature of COVID-19, and because of the respiratory difficulties of acutely ill COVID-19 patients, the use of only an inspiratory scan is preferred. However, PRM and DPM methodologies, used in the early stages of the mild-to-moderate disease or used to follow early interventional changes in the more normal lung regions, may still provide important quantitative information.

### Suggested protocol and imaging guidelines

The development and validation of qCT rely on standardized imaging protocols, as various technical factors like voxel size, image kernel can affect various parameters. As the CT technology is evolving, and there are multiple vendors with varying nomenclature, having a single universal protocol is not possible. However, if there is a similar principle for setting protocols which derive similar images between different vendors, quantitative lung imaging, and refined imaging data collection for AI or deep learning across vendors becomes possible. The principles for low dose qCT include:

1. Coaching the patient to full inspiration (total lung capacity).
2. Non-contrast scanning.
3. Use of 3D capable thin slices (to accommodate current clinical practices trying to limit the number of slices, we recommend 1.25 mm sections with 1 mm slice increments).
4. A quantitative image reconstruction kernels (avoiding sharp kernels which alter the HU at edges of structures and accentuate noise). In the case of Siemens, this is a Qr 40 or B30f, and for GE and Philips, this is ‘standard reconstruction’ algorithm.
5. Use of more modern iterative reconstruction algorithms (ADMIRE or SAFIRE on Siemens, ASiR-V on GE, iDose on Philips).
6. Use of the most current dose modulation implementations of the various manufacturers.
7. A coached full expiration [residual volume (RV) scan, if taking advantage of PRM and DPM analysis]. This technique has the potential to be relevant for longitudinal studies in COVID-19 survivors but may not be possible in acutely ill patients.

Table 1 summarize suggested CT imaging protocols for a single source and a dual-source Siemens, GE, and Philips scanner, respectively, based upon slight modifications to protocols developed for the more recent phases of several ongoing NIH-sponsored longitudinal multicenter studies, including SPIROMICS,^99^ MESA Lung,^100^ and PrecISE.^101^ Using the Siemens SOMATOM Force as the reference scanner, low-dose protocols have been established based upon the lowest dose, which does not interfere with the targeted airway and parenchymal metrics.^102,103^ The protocol was developed so as to achieve a common image noise and spatial resolution across scanner types as judged by imaging a modified anthropomorphic phantom produced by Kyoto Kagaku^104^ and inserts for assessment of density and modulation transfer functions produced by the Phantom Labs.^105^ A protocol for a scanner vendor or model not mentioned in Table 1 can be made using the above-stated principles for qCT lung scanning. Protocols that allow for consistent qCT metrics have been detailed in our previous work,^106^ but a minor difference is suggested in this protocol for COVID-19 to ensure integration with the existing clinical workflow. The slice thickness suggested is 1 mm (as compared to sub-mm), due to the technical challenges associated with a large number of CT images for clinical interpretation and storage requirements. The qCT metrics detailed in this work have been studied and validated on non-contrast images. Hence, if contrast-enhanced CT is performed for clinical indications (like rule out pulmonary embolism), the addition of low dose chest CT based on the suggested protocols will be useful for post-processing using qCT methods. Rapidly increasing literature highlights the clinical potential of qCT applications in COVID-19 pneumonia, and reiterates the need for standardization of lung CT imaging, such that these efforts can be applied to diverse populations, demographics, institutions, and scanner models.

**Table 1.** CT Chest protocols for quantitative lung imaging

	Siemens Definition AS+	Siemens FORCE	GE CT750 HD	GE Revolution	Philips iCT	Canon Genesis	Canon PrimeSP
Intravenous contrast	No	No	No	No	No	No	No
Detector configuration (number x mm)	128 × 0.6	192 × 0.6	64 × 0.625	128 × 0.625	128 × 0.625	80 × 0.5	80 × 0.5
SOV	–	–	Large body	Large body	–	Large	Large
Organ characteristic	Thorax *Ensured by first selecting a Siemens default routine adult chest protocol*	Thorax *Ensured by first selecting a Siemens default routine adult chest protocol*	–	–	–	–	–
Rotation time (s)	0.5	0,025	0.5	0.5	0.5	0.275	0.35
Scanner Pitch	1.0	1.0	0.984	0.992	1.0	Standard	Standard
kVp	120 (Automated kV control – off)	120 (Automated kV control – off)	120	120	120	120	120
Tube current modulation	Care dose: on	Care dose: on	AutomA & SmartmA: On	AutomA & SmartmA: On	DoseRight On	SureExposure On	SureExposure On
Quality reference mAs – Software v. 2012B, VA48, and VA50	42	36	–	–	–	–	–
Quality reference mAs – (VB10 & VB20)	29	25	–	–	–	–	–
Max mA	–	–	800		–	700	700
mA	–	–	–	SmartmA 30–500	–	20–700	20–700
Noise index	–	–	85	61	–	12.5	12.5
DRI	–	–	–	–	8	–	
Slice thickness x interval (mm)	1 × 0.5	1 × 0.5	1 × 0.5	1 × 0.5	1 × 0.5	1 × 0.5	1 × 0.5
Reconstruction Kernel	Q30f	Qr40	Standard	Standard	B (Standard)	Lung Standard	Lung Standard
Iterative algorithms	SAFIRE 5	ADMIRE 5	ASiR-V 60%	ASiR-V 60%	iDose 6	AIDR3De	AIDR 3De
Reconstruction mode	–	–	Helical Plus	Helical Plus	–	–	
Additional filters	–	–	IQ Enhance OFF	IQ Enhance OFF	–	–	
Approximate dose (for a 30 cm scan, average-size adult)	CTDIvol: 2.46 mGy Effective dose: 1.65 mSv	CTDIvol: 2.21 mGy Effective dose: 1.49 mSv	CTDIvol 4.87 mGy Effective dose: 3.27 mSv	CTDIvol: 3.38 mGy Effective dose: 2.27 mSv	CTDIvol: 3.5 mGy Effective dose: 2.35 mSv	CTDIvol: 2.4 mGy Effective dose: 1.61 mSv	CTDIvol: 2.4 mGy Effective dose: 1.61 mSv

CTDIvol, volume computed tomography dose index; DRI, dose right index ; SOV, scan field of view.

### Potential of qCT imaging research for COVID-19

Based on extensive data on multiple lung diseases described, early feasibility studies on qCT metrics among COVID-19 pneumonia patients, and cases shown in this practice and policy document, the use of previously validated CT acquisition protocols and qCT imaging has great potential for research allowing a better understanding of the disease. Quantitative lung CT metrics may have a role in identifying individuals that are at a higher risk of lung disease progression as compared to individuals that are at lower-risk and hence can be managed by home care. Other potential roles for qCT metrics include better characterization of disease phenotype and how treatment may be optimized for individual patients. Delineation of bronchocenteric disease patterns and their quantification in the sublobar segments using tMPR or summary views may enhance diagnosis and disease quantification. Due to heterogeneous nature of the COVID-19 disease with varied lung affection among patients, there is an ongoing debate as to whether distinct clinical phenotypes of COVID-19 exist based on respiratory compliance^107^ or CT imaging features, or if the lung disease is distributed along a continuum of severity similar to ARDS.^108,109^ Given such wide distributions in the severity of the disease, voxel attenuation characteristics on CT imaging, ventilatory responsiveness, and time between onset and clinical presentation, qCT can play a significant role in assessing the response to treatment early and objectively, and help resolve such debates. Newly emerging radiomics methods, combined with supervised ML, has been considered a next step in line with qCT in the field of oncology,^110–114^ whereby the growing list of qCT metrics yield disease insights. Beyond oncology, there are a growing number of pulmonary applications that employ ML methodologies to qCT-derived metrics to demonstrate patient clusters with common clinical characteristics.^50,115–123^ Use of qCT metrics for featurization upstream of ML algorithms has the potential to enhance the positive impact of AI on medical imaging.

### Summary

Imaging in COVID-19 pneumonia is challenging, but automated qCT can help beyond widely used qualitative subjective disease assessment. Quantitative lung imaging applications are fast and automated. They can help provide a reproducible objective assessment. Texture analysis can help to classify and quantify the radiological abnormality. Standardized lung CT acquisition is vital to apply qCT algorithms. Based on our experience in the role of qCT imaging in multiple other lung diseases, it is very likely that objective quantification of COVID-19 pneumonia and identification of its imaging phenotypes may have a role in diagnosis, patient triage, and predicting outcomes. The clustering of patients with common phenotypes and similar clinical outcomes may augment treatment decisions and validation of future clinical trials.
